# Unicompartmental knee arthroplasty and revision total knee arthroplasty have a lower risk of venous thromboembolism disease at 30 days than primary total knee arthroplasty

**DOI:** 10.1186/s43019-020-00078-9

**Published:** 2020-11-04

**Authors:** Andrew M. Schneider, Daniel R. Schmitt, Nicholas M. Brown

**Affiliations:** Department of Orthopaedic Surgery and Rehabilitation, Loyola University Medical Center, 2160 S. 1st Ave, Maywood, IL 60153 USA

**Keywords:** Total knee arthroplasty, Unicompartmental knee arthroplasty, Venous thromboembolic disease, Revision knee arthroplasty

## Abstract

**Background:**

While multiple studies have demonstrated a lower venous thromboembolism disease (VTED) risk for unicompartmental knee arthroplasty (UKA) compared to primary total knee arthroplasty (TKA), recent reports have shown that revision TKA also had a lower VTED risk compared to primary TKA, an unexpected finding because of its theoretical increased risk. Given the paucity of up-to-date comparative studies, our goal was to perform a high-powered VTED risk comparison study of UKA and revision TKA to primary TKA using recent data.

**Methods:**

The National Surgical Quality Improvement Program (NSQIP) database was queried between 2011 and 2018, and we identified 213,234 patients for inclusion: 191,810 primary TKA, 9294 UKA, and 12,130 revision TKA. Demographics, medical comorbidities, and possible VTE risk factors were collected. Thirty-day outcomes, including deep vein thrombosis (DVT), pulmonary embolism (PE), and all-cause VTED were compared between knee arthroplasty types.

**Results:**

On multivariate analysis, UKA was significantly associated with lower rates of DVT [OR 0.44 (0.31–0.61); *P* < 0.001], PE [OR 0.42 (0.28–0.65); *P* < 0.001], and all-cause VTED [OR 0.42 (0.32–0.55); *P* < 0.001] when compared to primary TKA. Revision TKA was significantly associated with lower rates of PE [OR 0.62 (0.47–0.83); *P* = 0.002], and all-cause VTED [OR 0.82 (0.70–0.98); *P* = 0.029] when compared to primary TKA.

**Conclusions:**

Utilizing recent data from a nationwide patient cohort and controlling for confounding variables, our results showed that both revision TKA and UKA had a lower risk of VTED compared to primary TKA, corroborating the results of recent investigations. Additional prospective investigations are needed to explain this unexpected result.

## Background

Venous thromboembolism disease (VTED), which includes both deep vein thrombosis (DVT) and pulmonary embolism (PE), is an inherent risk of lower-extremity-joint arthroplasty [1–3]. Though infrequent, the significant morbidity and mortality associated with VTED make a thorough understanding and mitigation of its risk essential [4–7]. As a result, evidence-based guidelines have been proposed in an effort to limit VTE events, while concurrently minimizing bleeding risk. In the most up-to-date iteration of its guidelines, the American Academy of Orthopaedic Surgeons (AAOS) recommended some form of prophylaxis via pharmacologic agents and/or mechanical compressive devices in patients undergoing hip and knee arthroplasty without an elevated bleeding or VTED risk [8]. However, this recommendation does not discriminate between arthroplasty types, despite differing reported rates and risks of VTED in the literature [9–11].

While there have been numerous studies examining the risk of VTED in primary total knee arthroplasty (TKA), far fewer studies have investigated how that risk compares to other knee arthroplasty procedures, such as unicompartmental knee arthroplasty (UKA) and revision TKA [1, 2, 12–17]. Given the paucity of reliable comparative studies comparing the VTED risk of UKA and revision TKA to primary TKA, we designed a large-scale, multivariate analysis using a national database with recent data. We hypothesized that UKA would have less risk of VTED and revision TKA would have greater risk of VTED due to their relative differences in surgical complexity compared to primary TKA.

## Methods

**Fig. 1:**
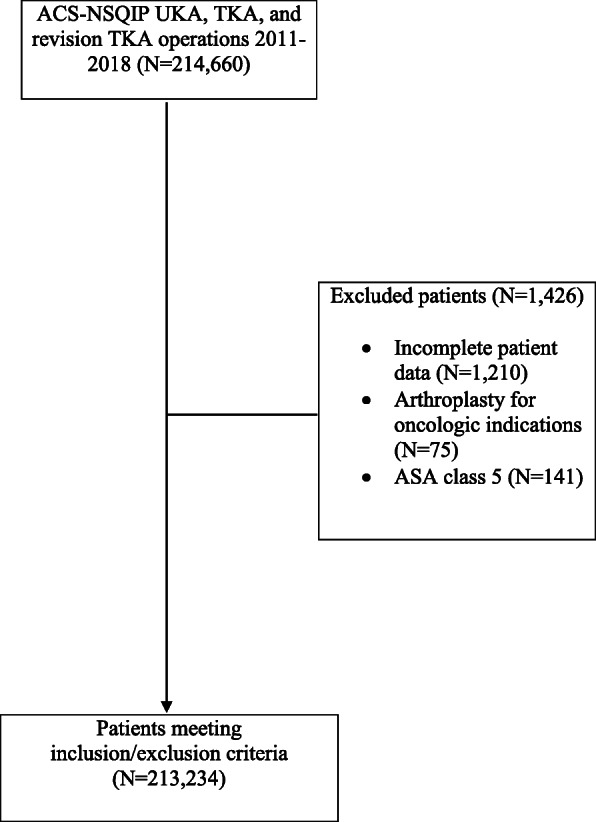
Flow chart demonstrating patient selection process

The study population was generated using the American College of Surgeons National Surgical Quality Improvement Program (ACS-NSQIP) database between the years of 2011 and 2018. The NSQIP is a validated outcomes database that reports on surgical outcomes in the 30-day post-operative period, from data gathered by trained surgical clinical reviewers from more than 700 hospitals across the United States. Its data has been found to be generalizable and extremely accurate, with rates of discrepancy < 2% on random audits [11, 18]. Inclusion criteria were patients identified using Current Procedural Terminology (CPT) codes 27,447 (arthroplasty knee, condyle and plateau; medial and lateral compartments with or without patellar resurfacing), 27,446 (arthroplasty, knee, condyle and plateau; medial or lateral compartment), and 27,486 and 27,487 (revision of TKA, with or without allograft, one component and revision of TKA, with or without allograft, femoral and entire tibial component, respectively). Exclusion criteria were incomplete patient data, arthroplasty for oncologic indications, and American Society of Anesthesiologists (ASA) class 5 patients. A flow chart demonstrating patient selection can be found in Fig. 1. This study was exempt from Institutional Review Board (IRB) approval because of the de-identified nature of the database.

Demographic variables including age, sex, and body mass index (BMI), as well as comorbidities including diabetes mellitus (DM), smoking status, anesthesia type, and ASA class were recorded for each patient. Operative time was also obtained. This study utilized the available data to determine the risk of DVT, PE, and all combined causes of VTED among the different knee arthroplasty types for the 30-day period following surgical intervention.

### Statistical analysis

The data analysis was completed using SPSS (V26, Armonk, NY, USA). This study conducted univariate analysis by dividing the subjects according to age (18–54, 55–75, and > 75 years), BMI (< 25, 25–35, 35–45, and > 45), and operative time (< 2, 2–4, and > 4 h) based on clinically relevant groupings. Univariate analysis was performed using chi-squared tests. Statistical significance was set at a *P* value < 0.05. Separate binary multivariate logistic regression models were built using DVT, PE, and all-cause VTED as outcome variables. These models included age, gender, BMI, ASA class, DM history, smoking history, anesthesia type, operative time, and type of knee arthroplasty.

## Results

Of the 213,234 patients included in our analysis, 191,810 (90.0%) underwent primary TKA, 12,130 (5.7%) underwent revision TKA, and 9294 (4.5%) underwent UKA.

On univariate analysis (Table 1), older patients had an increased incidence of DVT, PE, and all-cause VTED (*P* < 0.001), while patients with an increased BMI had an increased incidence of PE (*P* < 0.001) and all-cause VTED (*P* = 0.023). More women than men had a PE. General anesthesia (*P* = 0.003) and history of DM (*P* = 0.016) were associated with DVT. Patients undergoing UKA or revision TKA had a decreased incidence of DVT, PE, and all-cause VTE compared to TKA. Neither ASA class nor operative time were associated with DVT, PE, or all-cause VTED.

**Table 1:** Univariate analysis of demographic and comorbidity variables for deep vein thrombosis, pulmonary embolism, and all-cause venous thromboembolic disease

	DVT		PE		VTED	
	*N* (%)	*P* value	N (%)	*P* value	*N* (%)	*P* value
Age (years)						
18–54 (*N* = 23,636)	192 (0.8%)	**< 0.001**^a^	103 (0.4%)	**< 0.001**	274 (1.2%)	**< 0.001**
55–75 (*N* = 143,972)	1182 (0.8%)		815 (0.6%)		1858 (1.3%)	
> 75 (*N* = 45,626)	471 (1.0%)		323 (0.7%)		722 (1.6%)	
Body mass index (kg/m^2^)						
< 25 (*N* = 21,699)	189 (0.9%)	0.101	75 (0.3%)	**< 0.001**	244 (1.1%)	**0.023**
25–34.99 (*N* = 119,669)	1083 (0.9%)		681 (0.6%)		1638 (1.4%)	
35–44.99 (*N* = 60,374)	485 (0.8%)		419 (0.7%)		828 (1.4%)	
45+ (*N* = 11,492)	88 (0.8%)		66 (0.6%)		144 (1.3%)	
Sex						
Male (*N* = 82,759)	743 (0.9%)	0.203	421 (0.5%)	**< 0.001**	1090 (1.3%)	0.498
Female (*N* = 130,475)	1102 (0.8%)		820 (0.6%)		1764 (1.4%)	
History of smoking (*N* = 18,142)	160 (0.9%)	0.806	897 (0.5%)	0.062	229 (1.3%)	0.362
History of diabetes mellitus (*N* = 37,948)	289 (0.8%)	**0.016**	224 (0.6%)	0.826	486 (1.3%)	0.290
Anesthesia type						
Regional (*N* = 107,001)	862 (0.8%)	**0.003**	641 (0.6%)	0.319	1387 (1.3%)	0.083
General (*N* = 105,931)	982 (0.9%)		599 (0.6%)		1465 (1.3%)	
ASA class						
Class 1 (*N* = 4231)	40 (0.9%)	0.337	14 (0.3%)	0.075	51 (1.2%)	0.097
Class 2 (*N* = 106,509)	884 (0.8%)		600 (0.6%)		1366 (1.3%)	
Class 3 (*N* = 99,006)	892 (0.9%)		607 (0.6%)		1390 (1.4%)	
Class 4 (*N* = 3488)	29 (0.8%)		20 (0.6%)		47 (1.3%)	
Operative time (hours)						
< 2 (*N* = 172,154)	1467 (0.9%)	0.406	992 (0.6%)	0.066	2270 (1.3%)	0.117
2–4 (*N* = 33,384)	308 (0.9%)		189 (0.6%)		463 (1.4%)	
> 4 (*N* = 7696)	70 (0.9%)		60 (0.8%)		121 (1.6%)	
Arthroplasty type						
Primary total Knee arthroplasty (*N* = 191,810)	1706 (0.9%)	**< 0.001**	1169 (0.6%)	**< 0.001**	2655 (1.4%)	**< 0.001**
Unicompartmental knee arthroplasty (*N* = 9294)	36 (0.4%)		22 (0.2%)		52 (0.6%)	
Revision total knee arthroplasty (*N* = 12,130)	103 (0.8%)		50 (0.4%)		147 (1.2%)	

^a^Bold type signifies statistical significance, *P* < 0.05

On multivariate analysis (Table 2), UKA was found to be significantly associated with lower rates of DVT [OR 0.44 (0.31–0.61); *P* < 0.001], PE [OR 0.42 (0.28–0.65); *P* < 0.001], and all-cause VTED [OR 0.42 (0.32–0.55); *P* < 0.001] when compared to primary TKA. Additionally, revision TKA was found to be significantly associated with lower rates of PE [OR 0.62 (0.47–0.83); *P* = 0.002], and all-cause VTED [OR 0.82 (0.70–0.98); *P* = 0.029] when compared to primary TKA.

**Table 2:** Multivariate logistic regression for risk of deep vein thrombosis, pulmonary embolism, and all-cause venous thromboembolic disease among primary total knee arthroplasty, unicompartmental knee arthroplasty, and revision total knee arthroplasty

	Deep vein thrombosis			Pulmonary embolism			Venous thromboembolic disease		
	%	Odds ratio (95% CI)	*P* value	%	Odds ratio (95% CI)	*P* value	%	Odds ratio (95% CI)	*P* value
Primary total knee arthroplasty (*N* = 191,810)	0.9	Ref.^a^	–	0.6	Ref.	–	1.4	Ref.	–
Unicompartmental knee arthroplasty (*N* = 9294)	0.4	0.44 (0.31–0.61)	**< 0.001**^**a**^	0.2	0.42 (0.28–0.65)	**< 0.001**	0.6	0.42 (0.32–0.55)	**< 0.001**
Revision total knee arthroplasty (*N* = 12,130)	0.9	0.91 (0.74–1.12)	0.39	0.4	0.62 (0.47–0.83)	**0.002**	1.2	0.82 (0.70–0.98)	**0.029**

Bold type signifies statistical significance, *P* < 0.05

^a^Ref. is the baseline reference procedure to which all other procedures are compared

## Discussion

In the current study, we compared the VTED risk of UKA and revision TKA to primary TKA using a high-powered national database with recent data in an effort to corroborate the existing literature on this topic. After controlling for confounding variables, both UKA and revision TKA had a lower risk of VTED compared to primary TKA. Despite advancements in the understanding of perioperative, lower-extremity arthroplasty risks and mitigation practices put forth, serious complications, such as VTED, still occur. Therefore, it is imperative to continually evaluate these risks. Recent reports have consistently shown a lower VTED risk in UKA compared to primary TKA [19–21]. While it has been shown that revision TKA has a higher overall complication risk, including systemic sepsis and deep incisional surgical site infection, there have been few reliable studies using recent data that have investigated the comparative VTED risk of TKA and revision TKA specifically [22].

The lower VTED risk for UKA compared to primary TKA found in our study is supported in the literature. In a report of 423 consecutive UKA patients, Lombardi et al. reported no symptomatic VTED events [14]. In a 2019 study by Hansen et al., over 20,000 UKA and 400,000 TKA patients were identified in two large nationwide databases and followed for 90 days post surgery [21]. To control for any selection bias, propensity score matching was used on UKA and TKA patients and UKA patients were subsequently found to have a significantly lower risk of both DVT and PE [21]. Brown et al. performed a multi-institutional study looking at the incidence of postoperative complications [23]. Using 2235 primary TKAs and 605 UKAs performed at three institutions over a 5-year period, they found an increased incidence of VTE (1% vs 0.64%) in the TKA group, even after controlling for possible confounding variables, though this result was not statistically significant [23]. The lower VTED risk of UKA compared to TKA was an expected finding of our study. Compared with a primary TKA, a UKA requires less soft-tissue dissection, less hardware implantation, a shorter operative time, and faster recovery time [21, 24, 25]. Table 3 summarizes the recent literature comparing VTED risk in UKA and primary TKA [19–21, 23].

**Table 3:** Recent studies on comparative VTED risk between UKA and primary TKA

Study	Year(s)	Design	Follow-up	Unicompartmental knee arthroplasty				Primary total knee arthroplasty			
				*N*	DVT%^b^	PE%^c^	VTED%^d^	*N*	DVT%	PE%	VTED%
Brown	2004–2009	Retrospective	90 days	605	–	–	0.6	2235	–	–	1.0
Drager	2011–2012	Retrospective	30 days	1340	0.5	0.0^a^	–	36,274	0.9	0.7	–
Duchman	2005–2011	Retrospective	30 days	1588	0.5^a^	0.13	–	27,745	1.5	0.47	–
Hansen	2002–2012	Retrospective	90 days	20,488	2.04^a^	1.56^a^	–	415,727	3.70	3.01	–

*DVT* deep vein thrombosis, *PE* pulmonary embolus, *TKA* total knee arthroplasty, *UKA* unicompartmental knee arthroplasty, *VTED* venous thromboembolism disease

^a^Indicates significant difference compared to corresponding TKA value, *P* < 0.05

^b^Deep vein thrombosis percentage

^c^Pulmonary embolism percentage

^d^Venous thromboembolic disease percentage

The lower VTED risk for revision TKA compared to primary TKA found in our study is also supported in the literature. Bohl et al., using NSQIP data from 2011 to 2013, compared adverse events in primary versus revision total hip arthroplasty (THA) and TKA at 30 days post surgery [22]. They found that revision TKA had a lower incidence of both DVT and PE, though the risk was not significantly different from primary TKA. In a retrospective study from 2000 to 2011 of patients taking either aspirin or warfarin for chemoprophylaxis after total joint arthroplasty, Parvizi et al. found that the incidence of symptomatic PE was 1.4% in revision TKA and 1.8% in primary TKA [1]. In the only study to our knowledge that solely compares revision and primary TKAs, Boylan et al. retrospectively analyzed the New York Statewide Planning and Research Cooperative System (SPARCS), utilizing over 225,000 primary and revision TKAs from 2003 to 2012. They found the 30-day and 90-day risk of VTED to be lower for revision TKA than for primary TKA after controlling for age, race, gender, and medical comorbidities [16]. Of note, the data was geographically limited to one state, and smoking status, BMI, and operative time, previously cited risk factors for VTE, were not controlled for [26–28]. The lower VTED risk of revision TKA compared with primary TKA found in our study was not expected. Compared to primary TKAs, revision procedures are typically characterized as more complex. Aspects of revisions such as wider exposures, more soft-tissue disruption, increased volume and pressurization of cement, instrumentation of the medullary canals, and decreased post-operative mobility all theoretically may lead to an increased risk of VTED [11, 15, 16, 22]. However, reasons for findings to the contrary with respect to revision TKA, both in our study and in the recent literature, are still unclear. Patients with a VTE event during the primary TKA may be less likely to proceed with revision, or they might be put on a more aggressive thromboprophylactic regimen [16]. Additionally, frailer, less functional patients who are more prone to DVT may also be less likely to undergo aseptic revision. Further investigation to better understand this unexpected result is needed. Understanding the results of this study in the context of its limitations is critical. First, it is a retrospective review of a national database that tracks complications, reoperation, and readmission information, making it challenging to understand specific patient characteristics and factors that influence treatment. Moreover, post-operative anticoagulation regimens and overall patient mobility, including physical therapy protocols, are not elucidated in the database. Revision TKA is a very heterogeneous procedure, and the inevitable variability in the chemoprophylactic regimens could affect rates of VTED. Surgeons may use more aggressive regimens for revision cases as these are perceived to have a higher VTED risk based on traditional thinking: thus, VTED rate may be impacted. Variability in weight-bearing status post-operatively affects mobilization, putting patients with restricted weight-bearing at higher risk of VTED. Additionally, there likely exists a cohort of patients with prior VTED who are deemed too high-risk to undergo revision TKA; these patients remain unaccounted for in the current study. Lastly, the data on outcomes in the NSQIP database is limited to 30 days post surgery [11, 18].

## Conclusion

Utilizing recent data from nationwide patient cohort and controlling for confounding variables, our results showed that both revision TKA and UKA had a lower risk of VTED compared to primary TKA, corroborating the results of recent investigations. Additional prospective investigations are needed to explain this unexpected result.

## Data Availability

The data that supports the findings of this study are available from the National Surgical Quality Improvement Program (NSQIP) but restrictions apply to the availability of these data, which were used under license for the current study, and so are not publicly available. Data is, however, available from the authors upon reasonable request and with permission of the NSQIP.
